# A Conformal Tri-Band Antenna for Flexible Devices and Body-Centric Wireless Communications

**DOI:** 10.3390/mi14101842

**Published:** 2023-09-27

**Authors:** Wahaj Abbas Awan, Anees Abbas, Syeda Iffat Naqvi, Dalia H. Elkamchouchi, Muhammad Aslam, Niamat Hussain

**Affiliations:** 1 Department of Information and Communication Engineering, Chungbuk National University, Cheongju 28644, Republic of Korea; wahajabbasawan@chungbuk.ac.kr (W.A.A.); anees@chungbuk.ac.kr (A.A.); 2 Telecommunication Engineering Department, University of Engineering and Technology, Taxila 47050, Pakistan; iffat.naqvi@uettaxila.edu.pk; 3 Department of Information Technology, College of Computer and Information Sciences, Princess Nourah bint Abdulrahman University, P.O. Box 84428, Riyadh 11671, Saudi Arabia; dhelkamchouchi@pnu.edu.sa; 4 Department of Artificial Intelligence, Sejong University, Seoul 05006, Republic of Korea; 5 Department of Intelligent Mechatronic Engineering, Sejong University, Seoul 05006, Republic of Korea

**Keywords:** body-centric antenna, miniaturized antenna, muti-band antenna, flexible electronics, ISM, Wi-Max

## Abstract

A conformal tri-band antenna tailored for flexible devices and body-centric wireless communications operating at the key frequency bands is proposed. The antenna is printed on a thin Rogers RT 5880 substrate, merely 0.254 mm thick, with an overall geometrical dimension of 15 × 20 × 0.254 mm^3^. This inventive design features a truncated corner monopole accompanied by branched stubs fed by a coplanar waveguide. The stubs, varying in length, serve as quarter-wavelength monopoles, facilitating multi-band functionality at 2.45, 3.5, and 5.8 GHz. Given the antenna’s intended applications in flexible devices and body-centric networks, the conformability of the proposed design is investigated. Furthermore, an in-depth analysis of the Specific Absorption Rate (SAR) is conducted using a four-layered human tissue model. Notably, the SAR values for the proposed geometry at 2.45, 3.5, and 5.8 GHz stand at 1.48, 1.26, and 1.1 W/kg for 1 g of tissue, and 1.52, 1.41, and 0.62 W/kg for 10 g of tissue, respectively. Remarkably, these values comfortably adhere to both FCC and European Union standards, as they remain substantially beneath the threshold values of 1.6 W/kg and 2 W/kg for 1 g and 10 g tissues, respectively. The radiation characteristics and performance of the antenna in flat and different bending configurations validate the suitability of the antenna for flexible devices and body-centric wireless communications.

## 1. Introduction

Wearable and flexible electronics have gained significant attention in recent years in the industrial and academic worlds as they provide wide support for personal, Internet of Medical Things (IoMT), sports, military, and other applications [1,2,3]. IoMT applications can link intelligent devices to track vitals and performance, and to detect other health problems in patients. In order to establish a reliable wireless communication link, a body-centric antenna is required for wearable devices. Traditional antennas are not a popular choice for wearable applications due to their rigidness and inflexibility [4,5,6]. For wearable antennas, flexibility is required so that antennas can bend easily along with the body curves, providing comfort to the user. In addition to flexibility, it is desirable that wearable antennas must be light-weight, low-cost, maintenance-free, and small in size, as they have to be worn by the user [7,8].

In recent years, microstrip patch antennas have drawn considerable attention from researchers for wearable applications owing to their characteristics of light weight, low profile, ease of integration with electronic circuits, and less design complexity. Lately, several patch antenna designs have been reported in the literature for wearable devices covering the ISM (Industrial, Scientific, and Medical) band [9,10,11,12,13,14,15]. A variety of materials and structural designs have been investigated in these works to improve flexibility. The wearable antennas proposed in [9,10] are textile-based antennas for the 2.45 GHz ISM band. Similarly, PET, glass, transparent conductive-fabric polymer materials, flexible polyimide substrates, and cotton fabric materials have been used to produce wearable antennas [11,12,13,14,15]. Antennas reported in [10,11,15] exhibited good radiation characteristics; however, these designs have more design complexity due to their multilayered structures. Also, all these antennas are operating at single 2.45 GHz ISM band.

The rapid development of wireless technology requires communication systems to operate over multiple frequency bands supporting diverse applications. However, as compared to single-band and UWB antennas, it is challenging to design multiband antennas, as they require a specific impedance bandwidth within the desired frequency bands. Considering the importance of multiband antennas, several recent works reported wearable antennas operating at multiple frequency bands and utilized various substrates and design methodologies [16,17,18,19,20,21,22,23,24]. The antennas reported in [16,17,18,19,20] are dual-band antennas; however, these antennas possess comparatively large geometrical sizes. Also, metamaterial-inspired multilayered structures with dual-band operation are presented in [16,19], but this increases the design complexity. Similarly, another dual-band antenna with aperture-coupled multilayered fractal geometry has been reported for wearable devices [20]. In addition to dual-band antennas, tri-band wearable antennas have also been reported in the literature recently [21,22,23,24]. The works in [21,22,23] demonstrate antennas with layered designs utilizing the metamaterial, AMC, and multiple substrates design techniques to enhance the radiation characteristics of the antenna. Although the employment of these design techniques improved the antenna performance, it also increased the complexity and overall dimensions of the antenna design.

It is observed that the flexible antennas discussed above for wearable devices operating at dual- and triple-frequency bands have large dimensions as well as complex and multilayered structural designs. It is desirable that body-worn antennas are simple and compact in size. The large size of these antennas hinders their application for several body-worn devices. Considering the limitations exhibited by the works reported earlier, this work proposes a simple, compact, and flexible CPW-fed patch antenna for on-body and off-body wearable devices. This proposed antenna is realized on Rogers RT 5880 flexible substrate with 0.254 mm thickness and operates over the triple bands of 2.45/3.5/5.8 GHz for the ISM and WiMAX frequency bands. As body-worn antennas operate in close proximity to the human body, it is necessary to analyze the effects of electromagnetic wave exposure on the human body. For this purpose, a Specific Absorption Rate (SAR) analysis is also carried out for the proposed antenna, which demonstrates that SAR values meet the standards set by the FCC and European Union of a maximum acceptable SAR range of 1.6 W/kg for 1 g of tissue and 2 W/kg for 10 g of tissue. Also, a conformal analysis in both *x*- and *y*-axis ascertains good performance of the antenna, which proves the suitability of the proposed antenna for wearable devices.

## 2. Design Procedure of Proposed Work

**Figure 1:**
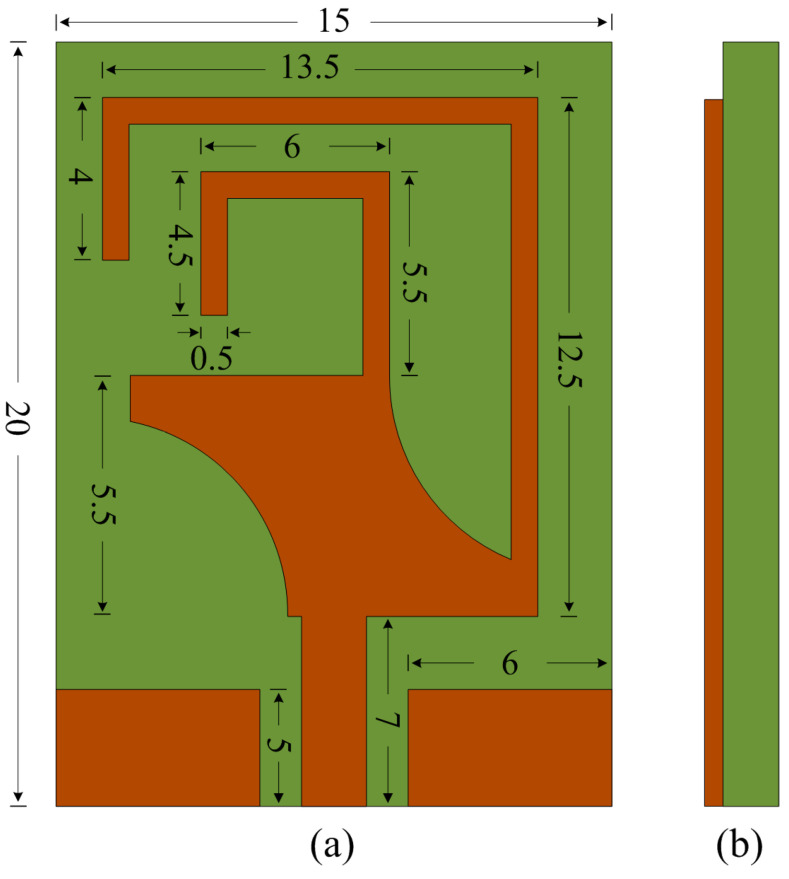
(**a**) Top and (**b**) side view of proposed work.

The design and simulation analysis of the proposed antenna were conducted using CST Studio. The proposed tri-band flexible antenna was modeled using the Rogers RT 5880 with a 0.254 mm thick substrate with dielectric constant of 2.2 and loss tangent of 0.0009. The antenna’s geometry was constructed on the topside of the substrate using standard copper cladding. A CPW-fed rectangular patch was modified using two semicircular slots etched from the top-right and bottom-left corner of the patch. Afterwards, a meandered line stub was loaded at the top-right corner to achieve the lowest band of 2.45 GHz while another stub was loaded at the middle of the patch to achieve a resonance of around 3.5 GHz. Impedance mismatch occurred due to the incorporation of the stubs, which was mitigated by optimizing the various dimensions of the antenna. The final design along with its optimized dimensions are shown in Figure 1.

### 2.1. Design Methodology

**Figure 2:**
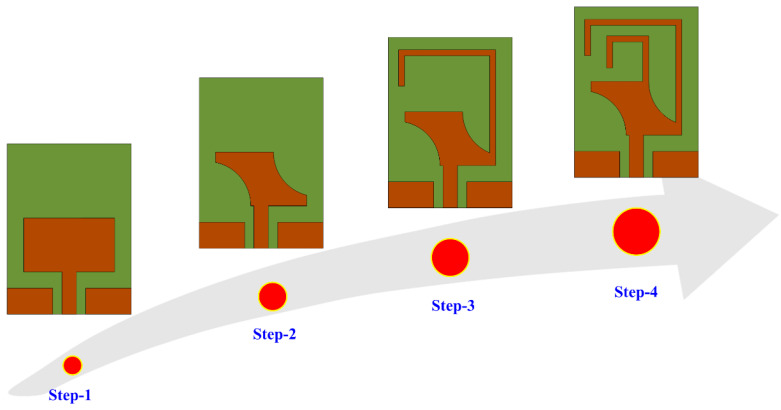
Design methodology staircase from basic to proposed antenna.

**Figure 3:**
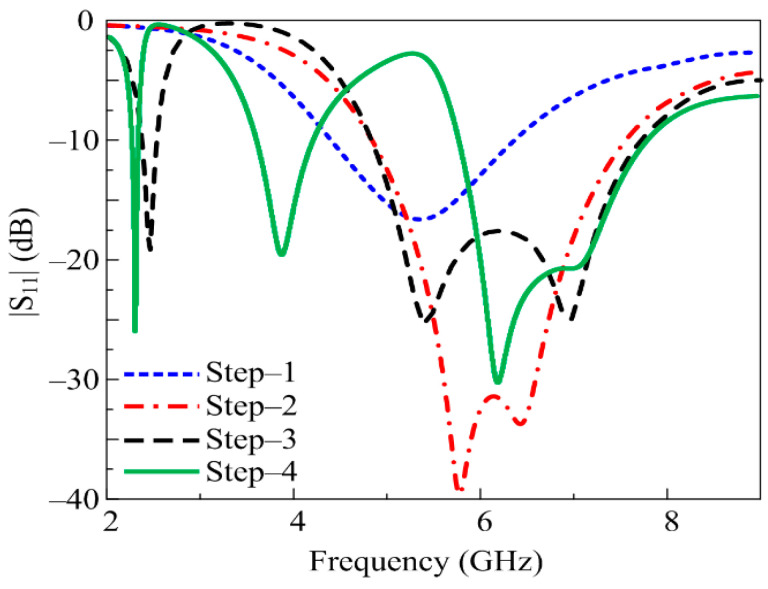
|S_11_| of various antennas included in design methodology.

The design methodology adopted to develop the proposed work is illustrated in Figure 2 (step-1 to step-4). The initial design consists of a CPW-fed rectangular patch antenna resonating at 5.8 GHz, as shown in Figure 2 (step 1). The mathematical expressions used to design the basic antenna for any desired frequency are widely available in the literature [25]. Afterwards, impedance matching was improved by modifying the design, which consequently increased the bandwidth of the antenna. For this purpose, a pair of semicircular slots were obtained by cutting the upper-right and lower-left corners of the radiator, as depicted in Figure 2 (step 2). The etching of these slots resulted in improved impedance matching and the return loss decreased from −15 to < −25, along with the bandwidth of the antenna also improving significantly from 2 GHz to 2.8 GHz, as shown in Figure 3. Later, in step 3, a meandered line stub was loaded at the top-right corner of the radiator. The insertion of this stub helped in achieving a low resonance of around 2.45 GHz. The effective length of the stub (*L_S_*) for any desired frequency (*f_d_*) can be estimated by using the following relationship:

(1) $$f_{d}=\frac{c}{tL_{s}\sqrt{\varepsilon_{ef}}}$$

where *c* denotes the light speed in free space, *t* denotes the fraction of free-space wavelength at the central frequency, while $\varepsilon_{ef\;}$ shows the effective dielectric constant of the substrate and can be estimated using the expression given below.

(2) $$\varepsilon_{ef}=\frac{\varepsilon_{r}+1}{2}+\frac{\varepsilon_{r}-1}{2}\{{\left(1+12\frac{S_{H}}{S_{W}}\right)}^{-0.5}+{0.04\left(1-\frac{S_{W}}{S_{H}}\right)}^{2}\}$$

$\varepsilon_{r\;}$ shows the dielectric constant, *S_H_* refers to the thickness of the substrate material, while *S_W_* represents the width of the substrate.

The total physical length of the inserted stub is 30 mm which corresponds to the quarter wavelength at the resonating frequency of 2.45 GHz. Figure 3 also validates the numerically estimated length of the stub. Similarly, for 3.5 GHz, the total length of the added stub in step 4 also corresponds to the quarter wavelength. However, it is noted in Figure 3 that insertion of a second stub not only introduced a new resonance at 3.5 GHz, but it also shifted the previously obtained bands from their desired ranges. Thus, further optimization is required to shift the frequency band to the required range.

### 2.2. Design Optimization

**Figure 4:**
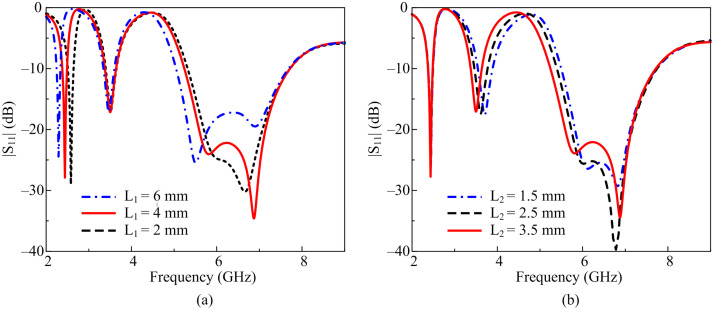
Parametric analysis of the open end of stubs: (**a**) larger stub and (**b**) shorter stub.

The shift in lowest resonance as well as the impedance mismatching at the higher resonance needed further optimization to cover the targeted bands and to achieve the desired results. For this purpose, a parametric sweep was carried out by tunning the total length of both stubs one by one, as shown in Figure 4. When the length of the longer stub was decreased from 4 mm to 2 mm, a shift in the lowest resonance was observed toward the higher side along with poor impedance performance at the highest resonating band, as shown in Figure 4a. Contrary to this, when the length of the stub was increased from 4 mm to 6 mm, the resonance shifted toward the lower side along with improved impedance matching at the higher region. Thus, by controlling the length of the larger stub, the lower resonance as well as the impedance matching at the highest passband can be optimized. It is interesting to note that the performance of the middle resonance remained unaffected during this optimization, as illustrated in Figure 4a.

Likewise, the parametric analysis of the shorter stub shows that changing the overall length of the stub resulted in a slight shift in the middle resonance from 3.5 GHz towards higher frequencies along with a decreased impedance-matching performance at the highest resonance. Meanwhile, the 2.45 GHz band remained unaffected while tuning the shorter stub, as illustrated in Figure 4b. This is proven by the results that the lower resonances are the consequent results of the tuning of the longer stub, while the impedance matching at the highest resonance depends upon the length of the shorter stub.

**Figure 5:**
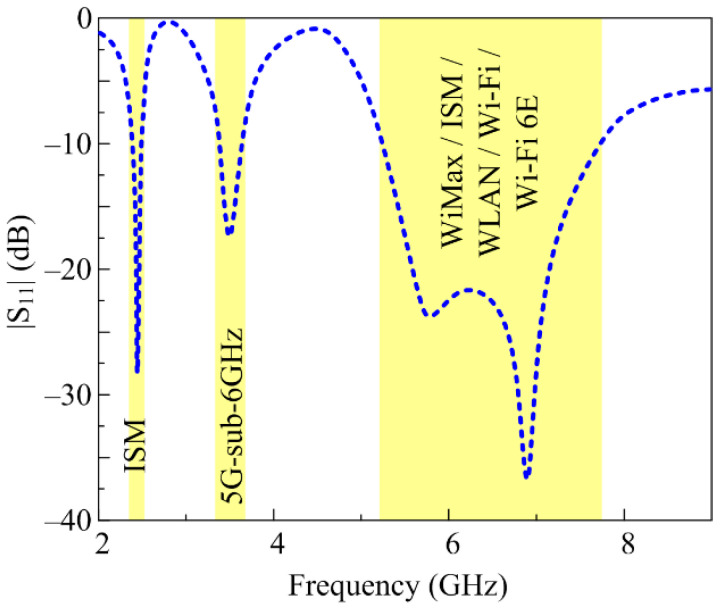
Optimized |S_11_| of the proposed tri-band antenna.

In this way, the antenna is optimized for the targeted band spectrum of 2.45 GHz, 3.5 GHz, and 5.8 GHz having |S_11_| < −10 dB impedance bandwidths of 2.37–2.5 GHz, 3.37–3.65 GHz, 5.2–7.7 GHz, respectively, as depicted in Figure 5. This shows that the proposed antenna can be used for a number of applications not limited to ISM, 5G sub-6-GHz, Wi-Max, Wi-Fi, WLAN, and Wi-Fi 6E.

### 2.3. Conformability Analysis

**Figure 6:**
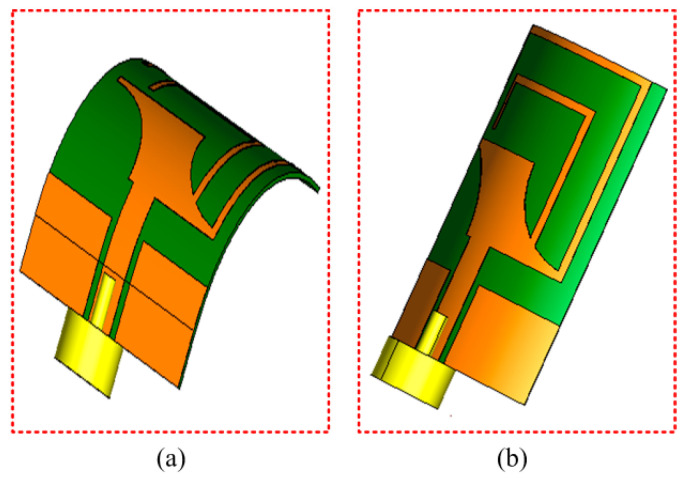
Simulation setup for conformability analysis when bending along (**a**) *x*-axis and (**b**) *y*-axis.

**Figure 7:**
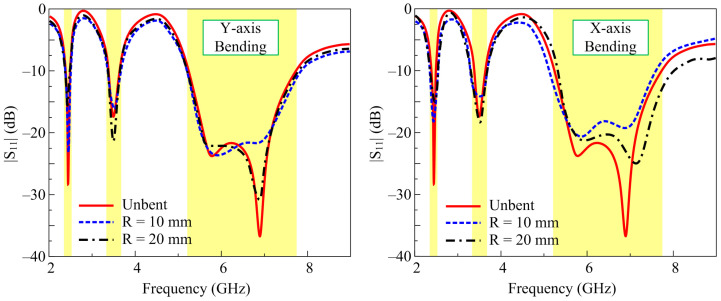
Simulated |S_11_| of the proposed work under conformable conditions.

As the proposed antenna is targeted to wearable devices supporting body-worn applications, it is therefore compulsory to verify the performance of the antenna in different conformal conditions [26]. Considering this, a confirmability analysis was carried out by bending the antenna in the *x*- and *y*-axis, at two radii of 10 and 20 mm, as shown by the simulation setup in Figure 6. The corresponding |S_11_| results for bent configurations at different axes and radii in Figure 7 exhibit non-significant alterations compared to the unbent configuration. Thus, the radiation characteristics of the antenna are not degraded when bent at different radii and axes.

## 3. Experimental Results

**Figure 8:**
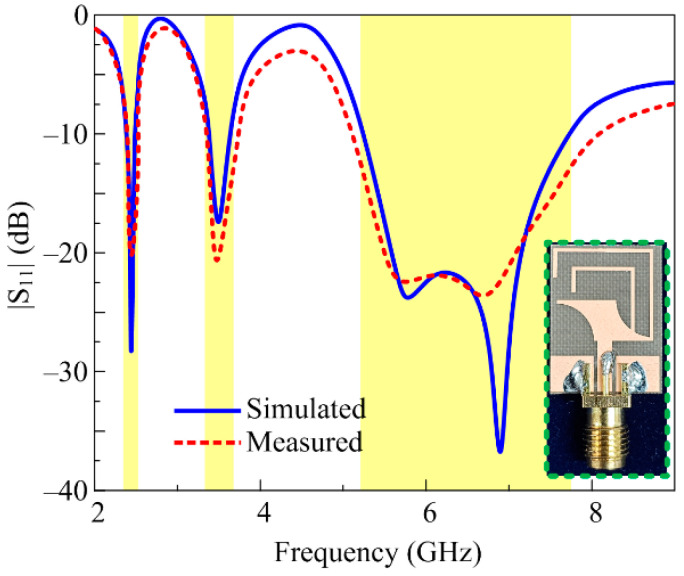
Fabricated prototype along with comparison of |S_11_| results.

In order to validate the results of the proposed antenna, experimental investigations were performed on the fabricated prototype, as illustrated in Figure 8. The subsequent sections provide a detailed analysis of the obtained simulated and measured results of the proposed antenna, as well the conformability analysis.

### 3.1. |S_11_|

A strong correlation is observed between the estimated and measured |S_11_| results due to the fact that the simulation model was excited using a 3D SMA-connector. The proposed antenna offers three resonances around 2.45, 3.5, and 5.8 GHz having an |S_11_| < –10 dB impedance bandwidth of 2.37–2.5 GHz, 3.37–3.65 GHz, and 5.2–7.7 GHz, which correspond to the percentage bandwidths of 5.3%, 8%, and 43%, respectively. It is important to note here that the proposed work covers the band spectrums of ISM-band (2.45/5.8 GHz), 5G sub-6-GHz (3.5 GHz), WLAN (2.4/3.6/4.9/5/5.9/6 GHz), Wi-Fi (2.4/5 GHz), and Wi-Fi 6E (6 GHz). Thus, this makes the proposed work a potential candidate for heterogenous applications requiring a multi-band antenna of compact size.

### 3.2. Radiation Pattern

**Figure 9:**
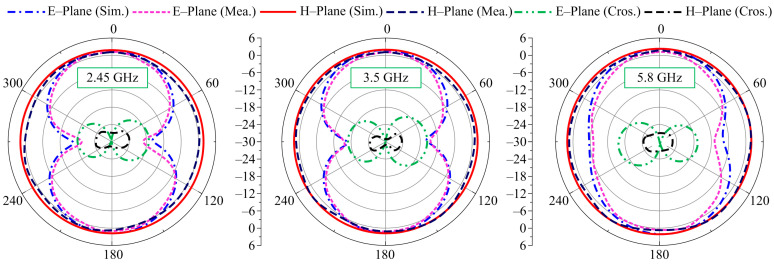
Predicted and measured radiation patterns at various frequencies.

The far-field behavior of the proposed tri-band antenna is observed at the selected frequencies of 2.45 GHz, 3.5 GHz, and 5.8 GHz, as depicted in Figure 9. The antenna offers an omni-directional radiation pattern in the H-plane for all selected frequencies where strong agreement is noted between the simulated and measured results. Moreover, the antenna possesses a similar pattern to that of a monopole in the E-plane at all selected frequencies, a bi-directional pattern in more concentrated and lower frequencies, while at higher frequencies the radiation pattern tends to show omni-directional-like characteristics. A slight variation among the predicted and measured results is a result of the measurement setup and fabrication process tolerance.

### 3.3. Gain and Efficiency

**Figure 10:**
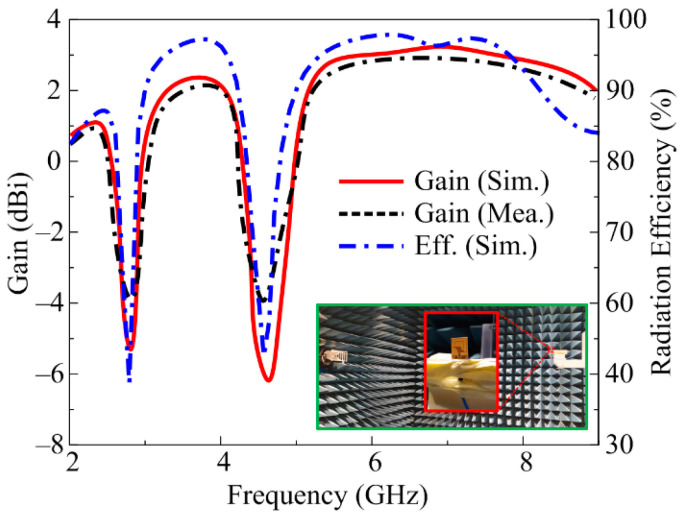
Gain and efficiency of proposed work along with far-field measurement setup.

The gain of the proposed antenna was also measured in an RF-isolated chamber by placing the antenna in front of a reference wideband horn antenna with standard spacing of 2 m, as shown in Figure 10. The antenna offers measured peak gains of 1.08, 1.96, and 2.99 dBi, while the simulated values show peak gains of 1.11, 2.01, and 3.03 dBi for the respective frequencies of 2.45, 3.5, and 5.8 GHz, as depicted in Figure 10. A radiation efficiency of more than 85% is observed for all passbands. Moreover, the gain as well as the efficiency of the antenna tend to decrease in non-resonating bands with minimum gains of −5.2 and −6.1 dBi and minimum efficiencies of 40% and 50% for the first and second non-operating regions, respectively.

### 3.4. Conformal Analysis

**Figure 11:**
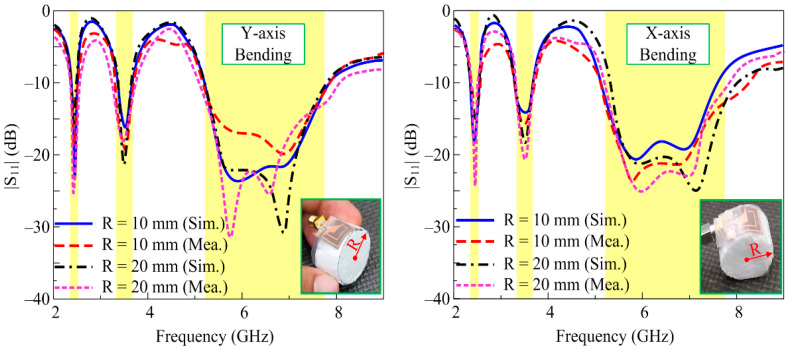
Simulated and measured |S_11_| for different bent configurations.

To analyze the performance of the antenna for wearable devices, a conformability test was also carried out. The antenna was bent along pieces of Styrofoam having radii of 10 mm and 20 mm in both the *y*- and *x*-axis, as depicted in Figure 11. As stated earlier, the simulated results did not differ notably in terms of s-parameter performance of the antenna in the bent configuration and under the unbent condition. A similar behavior was also observed when the antenna was measured with various radii in both axes. The strong correlation between the simulated and measured results in the conformal condition shows the potential of the presented work for flexible devices. The small amount of discrepancy observed among the results can be reduced by improving the fabrication accuracy as well as using a better measurement setup.

### 3.5. SAR Analysis

**Figure 12:**
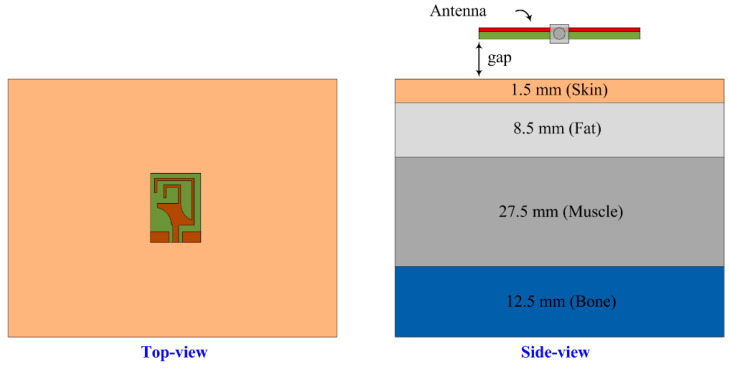
Setup utilized for the SAR analysis.

As wearable devices work close to the human body, the absorption of electromagnetic radiation by human tissue is hazardous. In order to ensure the safety of individuals, there are established standards for regulating the exposure of human tissues to electromagnetic (EM) radiation. These standards have been set forth by reputable organizations such as the International Commission on Non-Ionizing Radiation Protection (ICNIRP), the Institute of Electrical and Electronics Engineers (IEEE), and the Federal Communication Commission (FCC). These organizations play a crucial role in defining and enforcing guidelines to minimize the potential health risks associated with EM radiation exposure, thereby safeguarding human well-being. According to these regulatory bodies, human exposure at lower frequencies is evaluated in terms of SAR. Figure 12 shows the simulation setup of the proposed antenna for the SAR analysis. The antenna was placed on the topside of a four-layered human tissue model. The overall size of the human tissue model was 100 × 100 × 50 mm^3^. The antenna as placed with a gap between it and the tissue model, and the order of the layers along with their respective thicknesses are illustrated in Figure 12.

The permittivity and conductivity of the various layers are provided in Table 1 along with the respective SAR values with reference to 1 g and 10 g.

**Figure 13:**
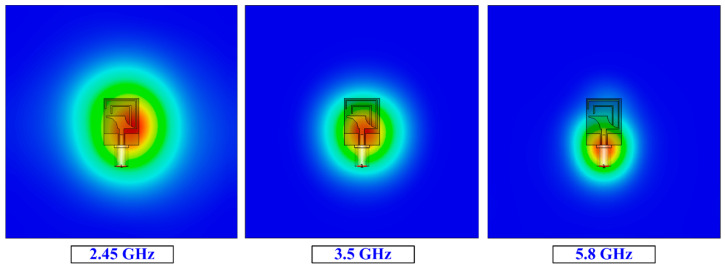
SAR analysis results for various frequencies.

In Figure 13, the results offered by the proposed antenna demonstrate SAR values of 1.48, 1.26, and 1.1 W/Kg for 1 g of tissue and 1.52, 1.41, and 0.62 W/Kg for 10 g of tissue at 2.45 GHz, 3.5 GHz, and 5.8 GHz, respectively. It is seen that all the values meet the threshold set by the FCC and other regulatory bodies with a maximum acceptable SAR range of 1.6 W/kg for 1 g of tissue and 2 W/kg for 10 g of tissue.

## 4. Comparison with State-of-the-Art Works

The flexible antenna proposed in this work is compared with recently published wearable antennas to further elucidate the merits of the proposed antenna. This comparison is summarized in Table 2. It is observed that the proposed antenna demonstrates better performance while maintaining structural simplicity and compactness in size. Although the antennas reported in [8,9,13] exhibit higher gain and lower SAR values, these antennas are operating at only a single band. Also, these antennas have larger dimensions and complex structures, which limit their suitability for wearable devices. Moreover, the works in [14,17,18,19,20,21] demonstrated dual- and triple-band wearable antennas; however, these antennas have large geometrical sizes. In addition, the design techniques used for these antennas increased the design complexity. Thus, the compact and simple structure of the proposed flexible antenna with tri-band operation and lower SAR values validates the suitability of this antenna for body-worn devices.

## 5. Conclusions

This paper introduces a compact and flexible antenna capable of operating in three frequency bands: 2.45 GHz, 3.5 GHz, and 5.8 GHz. These bands are significant for ISM (Industrial, Scientific, and Medical) and WiMAX applications. The antenna design incorporates a slotted rectangular patch configuration with a coplanar waveguide feedline. It is fabricated on a 0.254 mm thick Rogers RT 5880 substrate, with overall dimensions measuring 15 × 20 × 0.254 mm^3^. The primary goal of this antenna is to serve flexible and wearable devices within body area networks. To ensure its compatibility with such applications, the antenna’s flexibility is assessed through bending tests along both the x and y axes, at various radii. Additionally, an SAR analysis is conducted using a four-layered human tissue model to ensure safety. The obtained SAR values for the proposed antenna design at frequencies of 2.45 GHz, 3.5 GHz, and 5.8 GHz are determined as follows: 1.48 W/kg, 1.26 W/kg, and 1.1 W/kg for 1 g of tissue, and 1.52 W/kg, 1.41 W/kg, and 0.62 W/kg for 10 g of tissue, respectively. Importantly, all these values remain below the safety thresholds established by the FCC and European Union, which are 1.6 W/kg and 2 W/kg for 1 g and 10 g of tissue, respectively. Furthermore, the antenna’s radiation characteristics and performance are evaluated both in its flat and in various bending configurations. These assessments confirm the antenna’s suitability for wearable wireless communication devices, underscoring its effectiveness and real-world performance.

## Figures and Tables

**Table 1:** SAR analysis parameters and their respective values.

Frequency	Skin		Fat		Muscle		Bone		SAR (W/Kg) 1 g	SAR (W/Kg) 10 g
	*ε_r_*	σ	*ε_r_*	σ	*ε_r_*	σ	*ε_r_*	σ		
2.45	38.06	1.44	5.28	0.1	52.79	1.7	11.41	0.38459	1.48	1.52
3.5	36.84	2.59	5.069	0.2	50.91	2.9	10.46	0.67	1.26	1.41
5.8	35.114	3.71	4.9549	0.2	48.485	4.96	9.6744	1.544	1.1	0.62

**Table 2:** Comparison of the proposed antenna with other state-of-the-art works.

Ref.	Physical Size (mm^3^)	Electrical Size (λ^2^)	No. of Bands	Design Technique	SAR (W/Kg)	Gain (dBi)
[10]	45 × 45 × 2.4	0.37 × 0.37	Single band	High-impedance-surface-enabled design	0.0257	7.47
[11]	20.7 × 20.5 × 0.1	0.17 × 0.17	Single band	EBG-FSS	0.0182	7.9
[15]	122.5 × 122.5 × 1.8	1 × 4	Single band	AMC	0.371	7.2
[16]	70.4 × 76.14 × 3.11	0.57 × 0.62	Dual band	Metamaterial-based complementary split-ring resonator	0.381	3, 1.53
[19]	48.7 × 42.8 × 0.787	0.4 × 0.35	Dual band	Metamaterial-based split-ring resonator	1.79	2.7, 2.35
[20]	64.36 × 76.96 × 4.06	0.53 × 0.63	Dual band	Aperture-coupled fractal	1.24, 2.99	7.45, 4.75
[21]	28 × 32 × 0.394	0.23 × 0.26	Triple band	Metamaterial array	Not provided	2.5, 4.75
[22]	90 × 90 × 6	0.73 × 0.73	Triple band	AMC array	0.34	4.8, 5.1, 6.2
[23]	60 × 60 × 4.52	0.49 × 0.49	Triple band	Two-layered substrates	0.13, 0.09, 0.09	4.2, 6.6, 5.0
This work	15 × 20 × 0.254	0.12 × 0.16	Triple band	Stub-loaded patch	1.48, 1.26, 1.1 (1 g) 1.52, 1.41, 0.62 (10 g)	1.08, 1.96, 2.99

## Data Availability

All data are included in the study.
